# Risk Factors for Testicular Atrophy in Children With Testicular Torsion Following Emergent Orchiopexy

**DOI:** 10.3389/fped.2020.584796

**Published:** 2020-11-11

**Authors:** Xiao-Mao Tian, Xiao-Hui Tan, Qin-Lin Shi, Sheng Wen, Peng Lu, Xing Liu, Xu-Liang Li, Da-Wei He, Tao Lin, Guang-Hui Wei

**Affiliations:** ^1^Department of Urology, Children's Hospital of Chongqing Medical University, Chongqing, China; ^2^Ministry of Education Key Laboratory of Child Development and Disorders, Chongqing Key Laboratory of Pediatrics, National Clinical Research Center for Child Health and Disorders, China International Science and Technology Cooperation Base of Child Development and Critical Disorders, Children's Hospital of Chongqing Medical University, Chongqing, China; ^3^Chongqing Medical University, Chongqing Key Laboratory of Children Urogenital Development and Tissue Engineering, Chongqing, China

**Keywords:** pediatrics, testicular torsion (TT), atrophy, risk factors, urogenital system, urgent care

## Abstract

**Objective:** To analyze the risk factors for testicular atrophy (TA) in children with testicular torsion (TT) following emergent orchiopexy.

**Methods:** Clinical data of patients with TT undergoing orchiopexy were retrospectively reviewed, including age at surgery, affected side, delayed surgery (12–24 h and more than 24 h), echogenicity of testicular parenchyma on ultrasonography (ETPU), testicular blood flow on Color Doppler ultrasonography (CDUS), surgical findings (intraoperative blood supply, the degree of torsion, and surgical approaches), and follow-up. The primary outcome was the rate of TA after orchiopexy. The secondary outcome was the testicular volume loss (TVL) between the affected testis and the contralateral.

**Results:** A total of 113 patients were enrolled in this study with a median age of 11 years. The median follow-up was 21 months. Patients had a median TVL of 51.02% and 44 (38.94%) of them developed severe TA during follow-up. TA was significantly associated with age at surgery (*P* < 0.0001), delayed surgery (*P* = 0.0003), ETPU (*P* = 0.0001), and intraoperative blood supply (*P* = 0.0005). Multivariate logistic regression analysis showed that school-age children (OR = 0.069, *P* < 0.001) and puberty (OR = 0.177, *P* = 0.007) had a decreased risk of TA compared with preschool children, and that heterogeneous ETPU (OR = 14.489, *P* = 0.0279) and delayed surgery >24 h (OR = 3.921, *P* = 0.040) increased the risk of TA. Multivariate analysis demonstrated that ETPU (*F* = 16.349, *P* < 0.001) and delayed surgery (*F* = 6.016, *P* = 0.003) were independent risk factors for TVL.

**Conclusions:** Age at surgery, delayed surgery, and ETPU may play a crucial role in predicting the TA in children with TT following emergent orchiopexy. Moreover, blood flow measured by CDUS could not predict the outcome properly.

## Introduction

Testicular torsion (TT) has been considered as a serious surgical emergency that requires prompt diagnosis and surgical intervention. It is not only the most common cause of testicle loss in children but also may impair fertility and endocrine function of the testis in the future (1). The annual incidence of TT has been estimated to be around 0.004% among children <18 years (2). TT may occur at any age, but the vast majority of cases occur after age 10 years with a peak at 12 to 16 years (3). This disease features a dramatic reduction of testicular blood supply due to the torsion of the spermatic cord, with left-sided predominance, and rare bilaterality. The testicular torsion may be present intravaginally and extravaginally, and extravaginal torsion occurs more often in infants. Therefore, correct diagnosis and immediate treatment are crucial for the salvage when clinical assessments support or raise suspicion for spermatic cord torsion. Children and adolescents presenting acute scrotal pain for 12 h or more should still undergo surgical exploration because the viability of the testis is difficult to predict. Relevant research showed that testicular atrophy (TA) developed postoperatively in 12–68% of such cases, even though orchiopexy had been successfully performed (4–6). Most previous studies have focused on the predictors of intraoperative blood supply in the affected testis, mainly the degree and duration of torsion. However, no study has explored their combined influence on the prognosis of TT along with other factors. As such, the present study aimed to evaluate the occurrence of TA in children with TT following emergent orchiopexy and to identify risk factors involved in the development of testicular loss.

## Materials and Methods

### Patient Population

The protocol was approved by the local institutional review board. We identified all children with TT treated in the Children's Hospital of Chongqing Medical University between January 2009 and January 2019.

Clinical data were retrieved on age at surgery, affected side of testes, delayed surgery, echogenicity of testicular parenchyma on ultrasonography (ETPU), testicular blood flow on Color Doppler ultrasonography (CDUS), surgical findings, and follow-up. Inclusion criteria: TT confirmed by surgical exploration, successful orchidopexy, provision of informed consent by a parent or legal guardian. Exclusion criteria: age at surgery <2 years, follow-up <6 months or loss to follow-up, bilateral or congenital TT, cryptorchidism, testicular tumor, combined with systemic disease, or postoperative complications.

### Outcomes and Variables

The primary outcome was the rate of TA after orchiopexy. The secondary outcome was the testicular volume loss (TVL) between the affected testis and the contralateral. Patients were divided into 3 groups according to their age at surgery: preschool children (2–6 years), school-age children (7–12 years), and puberty (13–17 years). The delayed surgery was defined as the surgery more than 12 h after onset of pain. In addition, delays were further separated into two periods: 12–24 h and more than 24 h. CDUS findings included testicular size (L, length; W, width; H, height), ETPU, and testicular blood flow. The conditions of testes were evaluated based on the ETPU (homogeneous or heterogeneous), in which the testicular blood flow was either decreased or absent. Moreover, surgical findings consisted of intraoperative blood supply, the degree of torsion (<360° or more than 360°), and surgical approaches (orchidopexy or orchiectomy). Intraoperative rich blood supply was defined as the color of the affected testis changed to bright red; in contrast, poor blood supply meant that the color was maintained dark red.

### Management and Follow-Up

All operations were performed in a standard fashion. First, the affected testis was reset without tension. A moderate amount of 1% lignocaine was infiltrated within the spermatic cord. Second, the tunica vaginalis was opened to notice the color of the testis, the number of rotations, and the anatomy of the tunica vaginalis. The tunica albuginea was incised to lower intraparenchymal pressures. The exposed surface was covered with a piece of gauze moistened in warm saline and the blood supply was observed for 30 min. The contralateral testis was fixed with non-absorbable suture to reduce the risk of metachronous torsion. The affected testis was re-examined for potential viability, and the decision for orchidopexy or orchiectomy was made. Testicular infarct was characterized by coagulative necrosis of the testicular parenchyma, resulting in a diffuse reddening or blackening of the tissue. In this setting, surgical resection was taken into practice.

Patients had a routine follow up, with CDUS performed to evaluate the TVL between the affected testis and the contralateral. Testicular volumes were calculated using the modified ellipsoid volume formula, V = L^*^W^*^H^*^0.523. Currently, there is no standard definition of TA. We used a similar definition as suggested by relevant references (7, 8); TA was defined as the following: TVL ≥80% or no sustained blood supply in the follow-up.

### Statistical Analysis

Continuous variables and categorical variables were demonstrated as median value (range: min–max) and a percentage, respectively. Comparisons of variables between patients with and without TA were done with the chi-square test or Fisher's exact test for categorical data. Differences between groups were determined by non-parametric Mann–Whitney test (two groups) or Newman–Keuls test (multiple groups). Multivariate logistic regression analysis was used to examine the association of variables that were statistically significant in univariate analysis with TA, presented as odds ratios (OR) with 95% confidence intervals (95% CI). Multivariate analysis of variance was performed to evaluate the association between TVL and predictors. *P* < 0.05 were considered statistically significant. All analyses were performed using SPSS®, version 26.0 (IBM Corp., Armonk, NY, United States).

## Results

A total of 313 patients with TT were evaluated and 174 patients (55.59%) underwent orchiopexy. Of these, 128 patients had a complete follow-up. One child suffered continuous testicular enlargement at 5 months postoperatively, which was later diagnosed as a yolk-sac tumor by re-exploration, without any other complications such as infection or recurrence. Taking the effect of cryptorchidism on testicular development into account (9), we excluded 11 patients who had a history of cryptorchidism. Finally, a total of 113 patients were included after we identified some patients with other exclusion criteria. The median age at surgery was 11 years (range: 2–16) and the most frequently affected side of the testes was left-sided (85.84%). After a median follow-up of 21 months (range: 6–63), the patients in this cohort had a median TVL of 51.02% (range: 0–100), and 44 patients (38.94%) developed severe TA at their last visit.

As reported in Table 1, univariate analysis revealed that the following variables were significantly associated with TA: age at surgery (*P* < 0.0001), delayed surgery (*P* = 0.0003), ETPU (*P* = 0.0001), and intraoperative blood supply (*P* = 0.0005). Multivariate logistic regression analysis (Table 2) showed that school-age children (OR = 0.069, 95% CI: 0.018–0.256, *P* < 0.001) and puberty (OR = 0.177, 95% CI: 0.050–0.624, *P* = 0.007) had a decreased risk of TA compared with preschool children, and that heterogeneous ETPU (OR = 13.69, 95% CI: 2.424–77.31, *P* = 0.003) and delayed surgery >24 h (OR = 3.921, 95% CI: 1.061–14.486, *P* = 0.040) increased the risk of TA.

**Table 1:** Univariate analysis of testicular atrophy and testicular volume loss.

	**Normal testis**	**Testicular atrophy**	***P*-value**	**Testicular volume loss**	***P*-value**
	**(*n* = 69)**	**(*n* = 44)**		**Median (IQR)**	
Age at surgery (years)			<0.0001^*^		0.0372^*^
2–6	8 (7.08%)	26 (23.01%)	Ref	69.31 (48.98–90.51)	Ref
7–12	33 (29.20%)	8 (7.08%)	<0.0001^*^	26.80 (0.67–73.35)	0.0178^*^
13–17	28 (24.78%)	10 (8.85%)	<0.0001^*^	28.64 (9.70–90.28)	0.0495^*^
Side			0.5847		0.6080
Left	57 (50.44%)	40 (35.40%)		51.02 (10.76–87.65)	
Right	12 (10.62%)	4 (3.54%)		44.73 (1.48–85.92)	
Delayed surgery (hours)			0.0003^*^		<0.0001^*^
0–12	33 (29.20%)	8 (7.08%)	Ref	11.57 (3.61–42.24)	Ref
12–24	20 (17.70%)	10 (8.85%)	0.1860	62.29 (20.02–87.18)	0.0037^*^
>24	16 (14.16%)	26 (23.01%)	<0.0001^*^	78.40 (54.97–95.67)	<0.0001^*^
Degree of torsion			0.7201		0.7212
0–360°	40 (35.40%)	24 (21.24%)		54.21 (2.46–89.66)	
>360°	29 (25.66%)	20 (17.70%)		51.02 (12.04–76.14)	
ETPU^†^			0.0001^*^		<0.0001^*^
Homogeneous	27 (23.89%)	2 (1.77%)		12.03 (0.45–28.64)	
Heterogeneous	42 (37.17%)	42 (37.17%)		69.03 (13.68–91.18)	
Blood flow on ultrasonography			0.1146		0.5033
Decreased	18 (15.93%)	6 (5.31%)		54.58 (22.36–90.23)	
Absent	51 (45.13%)	38 (33.63%)		51.02 (6.30–87.65)	
Intraoperative blood supply			0.0005^*^		<0.0001^*^
Rich blood supply	48 (42.48%)	16 (14.16%)		17.82 (1.48–72.32)	
Poor blood supply	21 (18.58%)	28 (25.78%)		68.75 (51.02–92.87)	

*Data are expressed as median (IQR) or n (%) as appropriate*.

† *ETPU, Echogenicity of testicular parenchymal on ultrasonography*.

* *P < 0.05*.

**Table 2:** Multivariate logistic analysis of testicular atrophy.

**Variables**	**OR**	**95% CI**	***P*-value**
Echogenicity of testicular parenchymal on ultrasonography	13.69	2.424–77.31	0.003^*^
Intraoperative blood supply	0.844	0.264–2.692	0.774
**Age at surgery (control group: pre-school age)**			
School age	0.069	0.018–0.256	<0.001^*^
Puberty	0.177	0.050–0.624	0.007^*^
**Delayed surgery (control group: 0–12 h)**			
12–24 h	1.329	0.354–4.992	0.674
>24 h	3.921	1.061–14.486	0.040^*^

* *P < 0.05*.

Table 1 showed that the following variables were significantly associated with TVL: age at surgery (*P* = 0.0372), delayed surgery (*P* < 0.0001), ETPU (*P* < 0.0001), and intraoperative blood supply (*P* < 0.0001). The results in Table 3 indicated that 40.3% (*R*^2^ = 0.403) of the total variation in TVL could be explained by the regression model in multivariate analysis, in which ETPU (*F* = 16.349, *P* < 0.001) and delayed surgery (*F* = 6.016, *P* = 0.003) were independent risk factors for TVL.

**Table 3:** Multivariate analysis of variance of testicular volume loss.

**Variables**	**df**	***F***	***P*-value**
Echogenicity of testicular parenchymal on ultrasonography	1	16.349	<0.001^*^
Delayed surgery	2	6.016	0.003^*^
Age at surgery	2	1.780	0.174
Intraoperative blood supply	1	1.805	0.185

*R^2^ = 0.403*.

* *P < 0.05*.

## Discussion

TT is one of the most common causes of “acute scrotum” in children. Persistent ischemia can result in acute testicular damage, requiring immediate surgical intervention. However, testicular torsion-detorsion is an ischemia-reperfusion process of the testis. The injury induced by ischemia-reperfusion is notably more severe than the damage resulted from ischemia alone (10, 11). Besides, multiple theories were implicated to support the cross-injury theory and adverse effects on fertility, such as autoimmunization against the spermatogonia, decrease in testicular blood flow caused by a reflex sympathetic response, autoimmune reactions due to the disruption of the blood-testis barrier, and the generation of reactive oxygen species after detorsion (12). As with any medical treatment, the surgeon must weigh different complications associated with retention of testes (infection, malignant transformation, atrophy, and adverse effects on fertility) and removal of testes (trauma and psychosocial problems during puberty). While these concerns were acceptable preoperatively, the actual rate of orchiectomy after surgical detorsion was fairly high in general, and so was TA following emergent orchiopexy. Therefore, it is important to identify the preoperative or intraoperative factors related to prognosis, allowing emergency practitioners to figure out the correct surgical planning and the appropriate patient education. In this study, several potential risk factors for TA have been retrospectively evaluated, showing that age at surgery, delayed surgery, ETPU, and intraoperative blood supply were significantly correlated to the prognosis.

Delayed surgery and the degree of torsion were known prognostic factors for testicular viability during operation (13, 14). In a retrospective study, Lian et al. found that a higher incidence of postoperative TA was related to a delay of more than 24 h (6). Through a more detailed division of delay, the present study revealed that delayed surgery for more than 12 h increased significantly the TVL compared with the operation within 12 h and that delayed surgery for more than 24 h was significantly associated with an increased risk of TA. In the meantime, delayed surgery for more than 24 h was an independent risk factor for TA in the multivariate analysis after the effects of other variables were excluded. To the best of our knowledge, no other studies have demonstrated the association of the degree of torsion with TA. Compared with the incomplete twist of the spermatic cord, the complete twist (more than 360°) did not increase the risk of TA and demonstrated no significant reduction of testicular volume in our study, indicating the critical importance of timely surgical detorsion without preoperative clarifications about the degree of rotation. The intraoperative blood supply was another significant risk factor related to the prognosis but not an independent predictor.

CDUS offered a rapid, available, and safe modality in the diagnosis of TT to assess testicular architecture, intraparenchymal blood flow, and other anatomic details (hydrocele, scrotal thickening), with the advantages of non-invasive and repeatable examinations. But this study showed no significant correlation between blood supply on CDUS and outcomes. Nonetheless, CDUS showed accuracy in the imaging of affected testis with a high degree of sensitivity and specificity. We may pay attention to the heterogeneous ETPU in CDUS reports, which are signs of parenchymal edema or necrosis due to severe and persistent ischemia or hypoxia. This study found that TA and TVL could be predicted by heterogeneous ETPU, which was further confirmed by multivariate analyses. Another study also found that ETPU could predict testicular salvage after torsion (15). Based on these findings, emergency testis-sparing surgery could be performed for testis with homogeneous ETPU as soon as possible, to some extent, if no other risk factors were identified.

A previous study has reported a correlation between age at surgery and the rate of orchiectomy (16). However, the patients were not stratified based on age at surgery and no relevant investigations have found its influence on testicular prognosis. In our study, we realized that age at surgery was a predictor for the development of TA. Meanwhile, further analyses showed that the risk of TA reached a peak in the preschool period, which may be helpful in clinical decision-making and postoperative family counseling.

It has been reported that the outer membrane of affected testis was usually under high tension, due to obstruction of venous return and lymphatic circulation. This presentation resembles that of the testicular “compartment syndrome,” which might increase the possibility of testicular ischemia and necrosis. Previous studies concluded that a tunica albuginea fasciotomy to relieve compartment pressure followed by a tunica vaginalis flap may enhance salvage (17, 18). However, a major disadvantage of this technique is the higher rate of postoperative TA. The incision of tunica albuginea was adopted for decompression in our surgical procedures, yet this may impact spermatogenesis. On the other hand, the reliability of these techniques lacks large-scale validation at the moment. Of note, our intraoperative findings revealed that the rate of orchiectomy in our cohort was 44.41%. But if the proportion of patients with TA were included, more than 65% of all patients diagnosed with TT at our institution would ultimately develop severe testicular loss. Overall, surgical exploration without any delay remains the optimum strategy for improved outcomes.

Some principal limitations are noteworthy in our study. First, this was a retrospective, single-centered study, and so selection bias may exist. Secondly, CDUS is a subjective modality on the basis of a physician's experience in ultrasonic imaging, which may affect accuracy due to less consistency. Additionally, the follow-up was relatively short-term, and the absence of the examination of semen quality means that more atrophic testes and infertility are expected to occur as years come. It is possible that the torsed testicle of pre-pubertal boys, despite no TVL at present, will not develop during puberty and a significant volume loss may be appreciated later.

In summary, age at surgery, delayed surgery, and ETPU may play a crucial role in predicting the development of TA in children with TT following emergent orchiopexy. Blood flow on CDUS could not predict the outcome properly and intraoperative blood supply should not be the only foundation for the judgment of TA. Comprehensive assessments of these preoperative factors are necessary, which can be helpful for the surgeon to implement the correct surgical plan. If these indicators combined with intraoperative evaluation indicate a potential TA, the testis with a high risk of atrophy could be removed to avoid long-term complications. Otherwise, emergent surgery must be performed without any delay to save the testis.

## Data Availability Statement

The original contributions presented in the study are included in the article/supplementary material, further inquiries can be directed to the corresponding author/s.

## Ethics Statement

The studies involving human participants were reviewed and approved by the Institutional Review Board, Children's Hospital of Chongqing Medical University. Written informed consent to participate in this study was provided by the participants' legal guardian/next of kin. Written informed consent was obtained from the minor(s)' legal guardian/next of kin for the publication of any potentially identifiable images or data included in this article.

## Author Contributions

PL, XL, X-LL, D-WH, TL, and G-HW contributed to conception and design. XL, D-WH, TL, and G-HW contributed to administrative support. X-MT, Q-LS, and SW contributed to collection and assembly of data. X-MT, X-HT, and Q-LS contributed to data analysis and interpretation. X-MT and X-HT contributed to manuscript writing. All authors contributed to manuscript revision, read, and approved the submitted version.

## Conflict of Interest

The authors declare that the research was conducted in the absence of any commercial or financial relationships that could be construed as a potential conflict of interest.

## Abbreviations

TT: Testicular torsion
TA: Testicular atrophy
TVL: Testicular volume loss
CDUS: Color Doppler ultrasonography
ETPU: Echogenicity of testicular parenchyma on ultrasonography.
